# *In vitro* sensitivity of *Malassezia furfur* isolates from HIV-positive and negative patients to antifungal agents

**DOI:** 10.7705/biomedica.6871

**Published:** 2023-08-31

**Authors:** Kevin Ehemann, Andrés Contreras, Adriana Marcela Celis-Ramirez

**Affiliations:** 1 Grupo de Investigación Celular y Molecular de Microorganismos Patógenos, Departamento de Ciencias Biológicas, Universidad de los Andes, Bogotá, D. C., Colombia Universidad de los Andes Grupo de Investigación Celular y Molecular de Microorganismos Patógenos, Departamento de Ciencias Biológicas Universidad de los Andes Bogotá, D. C., Colombia

**Keywords:** Malassezia, HIV, antifungal agents, drug resistance, fungal, dermatitis, seborrheic, microbial sensitivity tests., Malassezia, HIV, antifúngicos, farmacorresistencia fúngica, dermatitis seborreica, pruebas de sensibilidad microbiana.

## Abstract

**Introduction.:**

*Malassezia* is a lipophilic and lipid-dependent yeast genus belonging to the skin microbiota of humans and other animals. However, due to dysbiosis processes or other factors in the host, this yeast can cause different pathologies, ranging from skin diseases, such as seborrheic dermatitis, to fungemia. Isolation of *Malassezia furfur* has been reported in HIV-positive patients with or without skin lesions. Due to its opportunistic nature and its variable resistance to antifungal compounds, it is relevant to know the *Malassezia* sensitivity profiles.

**Objective.:**

To determine the sensitivity to different antifungal agents, of clinical isolates of *M. furfur* obtained from HIV-positive or negative patients, with or without seborrheic dermatitis.

**Materials and methods.:**

Assessment of isolates sensitivity to itraconazole, voriconazole, fluconazole, and amphotericin B was performed by two techniques: (1) Broth microdilution using Clinical and Laboratory Standards Institute (CLSI) protocol M27-A3 with modifications; and (2) agar tests using Etest®.

**Results.:**

Isolates obtained from HIV patients showed an increase in the minimum inhibitory concentration of fluconazole, voriconazole, and amphotericin B, compared with those of non-HIV patients. Itraconazole was the antifungal with the lowest minimum inhibitory concentration (MIC) in most isolates.

**Conclusion.:**

We observed differences in the sensitivity profiles of *M. furfur* isolates according to the context of the patient. High MIC of antifungals like fluconazole, commonly used for treating pathologies caused by *Malassezia,* were identified.

## Resumen

**Introducción.:**

*Malassezia* es un género de levaduras lipofílicas que dependen de los lípidos y hacen parte de la microbiota de la piel de humanos y otros animales. No obstante, debido a procesos de disbiosis u otros factores en el huésped, esta levadura puede llegar a causar diferentes enfermedades: desde cutáneas (como dermatitis seborreica) hasta fungemias. Se han reportado aislamientos de *Malassezia furfur en* pacientes positivos para HIV, con lesiones cutáneas o sin ellas. Por su carácter oportunista y sensibilidad variable a los compuestos antifúngicos, es relevante conocer los perfiles de sensibilidad.

**Objetivo.:**

Determinar la sensibilidad a diferentes antifúngicos de aislamientos clínicos de *M. furfur* obtenidos de pacientes positivos o negativos para HIV, con dermatitis seborreica o sin ella.

**Materiales y métodos.:**

La sensibilidad de los aislamientos a itraconazol, voriconazol, fluconazol y anfotericina B, se determinó mediante dos técnicas: microdilución en caldo según el protocolo M27-A3 del *Clinical & Laboratory Standards Institute* (CLSI), con modificaciones, y pruebas en agar mediante Etest®.

**Resultados.:**

Los aislamientos obtenidos de pacientes con HIV mostraron aumento de la concentración inhibitoria mínima a fluconazol, voriconazol y anfotericina B, en comparación con los de pacientes sin HIV. Por otro lado, al evaluar la mayoría de los aislamientos, el itraconazol fue el antifúngico con la menor concentración inhibitoria mínima.

**Conclusión.:**

Se evidencian diferencias en los perfiles de sensibilidad de los aislamientos de *M. furfur,* según el contexto del paciente, y elevadas concentraciones inhibitorias mínimas de antifúngicos como el fluconazol, usados comúnmente para el tratamiento de las enfermedades causadas por *Malassezia* spp.

*Malassezia* is a genus of lipophilic and lipid-dependent yeast classified into 18 species, that are part of the skin microbiota in humans and other animals. Recently *M. auris, M. palmae* and *M. rara were* proposed ^1^^-^^4^. *Malassezia globosa, M. restricta,* and *M. sympodialis* are the most prevalent species in the human skin mycobiome ^5^^,^^6^. Despite being commensal, they can act as opportunistic pathogens causing atopic dermatitis, seborrheic dermatitis, folliculitis, and pityriasis versicolor, and can be involved in Crohn’s disease and pancreatic cancer ^7^^-^^9^. The pathophysiology of these processes is not entirely understood. However, it is related to an increase in the activity of lipases and phospholipases released by the yeast to obtain lipid compounds and produce biofilms, among others ^2^.

Topical antifungals such as ketoconazole or terbinafine are used to treat localized skin infections. In addition, in the case of inflammatory processes, the topical use of corticosteroid or calcineurin inhibitors is also required ^2^^,^^10^. If localized management fails, lesions persist or the extent is considerable, an oral-systemic antifungal such as itraconazole or fluconazole should be considered ^7^. On the other hand, new *Malassezia* growth inhibitor candidates could interfere with enzymes essential for its metabolism. Lysine is one of these candidates showing yeast growth inhibition *in vitro.* However, further studies are required ^11^. Other candidates include essential oils from different plants ^12^.

In medical practice, this type of skin pathology is diagnosed based on the patient’s clinical characteristics, and empirical management is given, so etiological isolation is not generally performed. It is important to highlight that these treatments require prolonged use of antifungal agents, which in turn takes the risk of leading to antifungal resistance development ^13^^-^^15^. Resistance mechanisms are not fully elucidated. Even so, biofilm formation, overexpression of iron-sulfur transporter such as ATM1 ^16^, different enzymes involved in the ergosterol biosynthesis pathway such as ERG5 ^17^ and ERG11 ^16^, and the presence of efflux pumps as the pleiotropic drug transporter PDR10 ^18^, are considered as possible yeast strategies to overcome the action of the antifungal agents.

Systemic infections by *Malassezia* spp. are also reported, predominantly in neonates or adults with some degree of immunocompromise (HIV infection, chronic corticosteroid use, cancer). Some related risk factors are using a skin catheter for lipid infusion or a central venous catheter, and prophylactic fluconazole ^6^^,^^19^^,^^20^. So far, *M. furfur, M. sympodialis,* and *M. pachydermatis* are involved with fungemia. In these cases, the available treatment with a high efficiency rate is amphotericin B ^6^. However, there is a report of an amphotericin B-resistant *M. sympodialis,* isolated from a neonatal intensive care unit, which was susceptible to voriconazole and fluconazole ^21^. Treatment duration varies depending on the causative species ^6^^,^^22^^-^^24^.

Considering immunosuppressive states as risk factors for local and systemic infection, previous studies have tried to identify differences in skin colonization by *Malassezia* spp. in patients with HIV infection known status. It has been demonstrated that seropositive patients have a higher concentration of *Malassezia* yeasts in the skin, both those with clinical manifestations of seborrheic dermatitis and those without lesions ^22^. A high percentage of patients with skin lesions had not yet started antiretroviral therapy (ARVT). These studies show an increased number of *M. furfur* isolates in seropositive compared with those of the seronegative population (16.7% versus 1.5%, respectively) ^24^^-^^26^.

Given the risk of resistance development, secondary to prolonged topical treatments of local skin infections, and *Malassezia’*s capability to cause systemic disease with fungemia, it is crucial to determine its sensitivity and resistance profiles. However, the Clinical and Laboratory Standards Institute (CLSI) M27-A3 *in vitro* reference method for yeast by microdilution has not been standardized for this lipid-dependent microorganism. Therefore, no official cut-off points exist to determine the sensitivity or resistance profiles to antifungals, albeit there are epidemiological cut-off values for *M. pachydermatis* and *M. furfur*^27^. For this reason, several authors have modified the M27A3 protocol by adding compounds for *Malassezia* growth and evaluating the minimum inhibitory concentration (MIC) ^27^^-^^32^. These studies reported resistance to azoles, particularly fluconazole, and to polyenes like amphotericin B, which showed higher levels of MIC. However, variable results in isolated species of systemic infections depend on the medium used in the tests ^6^^,^^33^.

The epsilon test (Etest®) technique is a well-established method for *Candida* spp. However, MIC determination is not standardized for *Malassezia* spp. Previous sensitivity studies reported the use of different culture media and lipid supplements finding varied sensitivity profiles for *M. pachydermatis* and *M. furfur.* These assessments showed that Etest® is equivalent in MIC to microdilution in *M. pachydermatis* and to some antifungal azoles for *M. furfur*^15^^,^^28^^-^^30^, but more studies are needed to reach this conclusion. In Colombia, only a few studies have been carried out with this methodology ^15^.

Considering all the above, it is important to establish a standard protocol to determine the antifungal resistance of *Malassezia* spp. In addition, considering the ease of assaying sensitivity through an Etest® compared to microdilution, it is appropriate to perform a study about concordance between both methods in species that may cause cutaneous and systemic infections.

To provide helpful information contributing to the knowledge about the sensitivity profile of *Malassezia* spp., we evaluated its sensitivity *in vitro* to commonly used antifungal therapeutic agents. We used *M. furfur from* previous isolates of HIV-positive and negative patients, with and without seborrheic dermatitis. The evaluation was done with two methods: broth microdilution and the Etest® methods. The concordance between the methods determined the Etest® as an alternative method with significant advantage in terms of its essential and categorical agreement with the microdilution method.

## Materials and methods

### Isolates and inoculum preparation

The isolates were obtained from the collection of microorganisms of the *Grupo de Investigación Celular y Molecular de Microorganismos Patógenos* (CeMoP) at the *Universidad de los Andes.* The strains were previously isolated from HIV-positive and negative patients, with and without seborrheic dermatitis (table 1) ^26^. The isolates were identified by their assimilation capacity of Tween 20, 40, 60, 80 and cremophorEL; and molecular typing by sequencing of 5.88 rDNA-IT2 followed by phylogenetic analyses ^26^. The inoculum was done by taking five colonies, adding them to 5 ml of 0.5% Tween 80 solution, performing homogenization by vortex and filtration with sterile gauze. The concentration of the solution was calculated using a Neubauer chamber and adjusting it to obtain a standard inoculum concentration of 2x10^6^ CFU/ml ^15^^,^^29^^,^^32^^,^^34^.

**Table 1:** *Malassezia furfur* isolates used from CeMoP research group

Clinical entity	Strains
No lesion (NL)	58.42 NL; 103.76 NL; 110.80 NL; 9.2 SL; 46 NL
Seborrheic dermatitis (SD)	57.41 SD; 115.84 SD; 2.1 SD; 9.1 SD; 11.1 SD
No dermatitis and HIV (HIV)	23.13 HIV; 90.64 HIV; 99.72 HIV; 117 HIV; 40.26A HIV
Seborrheic dermatitis and HIV (SDHIV)	24.14 SDHIV; 26.16 SDHIV; 29.18 SDHIV; 67.51 SDHIV; 37.248 SDHIV

### Broth microdilution test

The broth microdilution method was based on the protocol M27-A3 established by the CLSI ^34^. This technique is standardized for *Candida* spp. and *Cryptococcus* spp. We made modifications to achieve the growth of *Malassezia.* The culture medium used was Sabouraud supplemented with 0,5% Tween 40 and 0,5% Tween 60. Previous studies used this medium and showed adequate growth and easy visual evaluation of the results ^11^^,^^15^.

The solutions of the antifungals itraconazole, ketoconazole, voriconazole, and amphotericin B were prepared in 1% of dimethyl sulfoxide (DMSO) using serial double dilutions to obtain final concentrations ranging from 0.03 to 16 μg/ml. Fluconazole was prepared in sterile distilled water to final concentrations of 0.12 to 64 μg/ml ^34^.

The protocol was carried out in triplicate. Each plate was incubated at 33 ºC for 72 hours, and the MIC was checked every 24 hours using an inverted mirror. In the azole group, it was calculated at the point of a 50% decrease in growth concerning the control. Amphotericin B MIC was defined as the one at which no growth was evident ^34^.

### *Etest* ^*®*^ *assay*

It was necessary to standardize the growth medium to compare the two sensitivity methods. We used Sabouraud dextrose agar supplemented, the same way for microdilution, with 0.5% Tween 40 and 0.5% Tween 60. The inoculum previously adjusted to a final concentration of 2 x 10^6^ CFU/ ml was homogenized on the surface of the medium with a cotton swab on the agar. The antifungals itraconazole, voriconazole, and amphotericin B were evaluated at a concentration of 0.02-32 μg/ml and fluconazole between 0.16 and 256 μg/ml ^15^^,^^27^^,^^29^. We did not test ketoconazole strips as they were out of existence when the experiments were performed. Cultures were incubated for 72 hours at 33 °C. After that, we determined the MIC. This protocol was performed in triplicate.

### Data analysis

Essential and categorical agreement analyses were carried out to verify the performance of the Etest® against the microdilution test as the gold standard method. The essential agreement is defined when an isolate has MICs within plus or minus one doubling dilution using both methods. In contrast, the categorical agreement occurs when an isolate has the same category result *(i. e.* sensible or resistant) using both methods ^35^. Concerning the essential agreement analysis, the mean values of every MIC for each isolate were converted to Log_2_ base so the comparison could be performed as follows:

*Log*_*2*_ *MIC*_*Etest*_ *-Log*_*2*_ *MIC*_Microdilution_

If this subtraction results equal or less than 1 and equal or greater than -1, there is essential agreement because the MIC difference between both methods is within the ± 1 range.

In the case of the categorical agreement, no consensus was found on the epidemiological cut-off values for *M. furfur* isolated from skin, so we considered those proposed by previous studies for voriconazole, fluconazole, and amphotericin B ^27^^,^^36^. The tentative epidemiological cut-off values were obtained by determining the MICs of 78 *M. furfur* strains to these antifungals. Sensitive strains encompassed 95% of the evaluated isolates and resistant strains were those having a two-fold dilution higher than the modal MICs values. To conclude whether the categorical agreement analysis shows concordance, a Fisher test was performed based on the previously stated classification among sensitive or resistant isolates for both methods.

Regarding the relationship between the isolate MICs and the patient’s health condition (HIV status and dermatitis condition), we considered only the data from the validated method (microdilution). Shapiro-Wilk test was performed to assess data normality. We applied eight mean differences tests for each antifungal to determine if any of the mentioned conditions were linked to higher resistance of *M. furfur* clinical isolates. MIC data were converted to log_2_ base as before.

## Results

**Figure 1:**
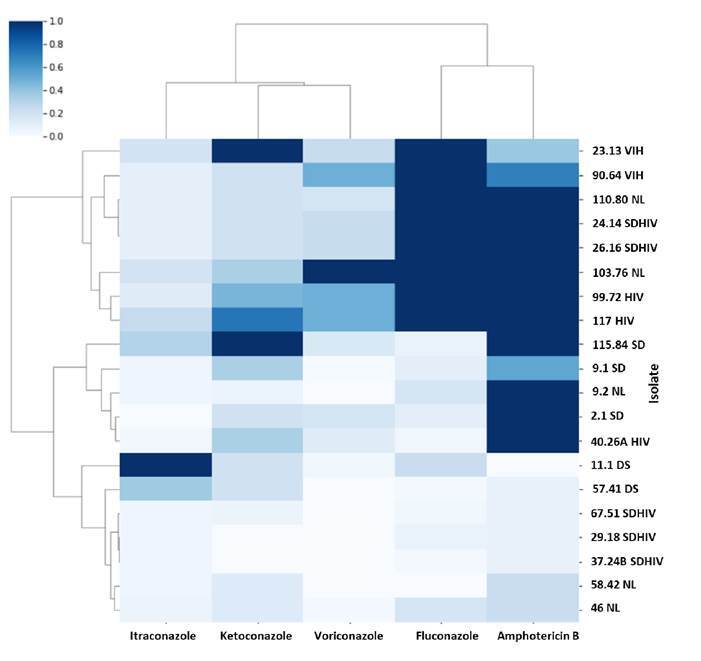
Heat map showing the sensitivity profiles for each antifungal agent (each one is normalized: 1 indicates the highest MIC value obtained and 0 the lowest value). Samples are grouped by similarity in their patterns. Three large groups are evident: ^1^ multiple resistance to fluconazole, amphotericin B and some to voriconazole, ^2^ resistance to amphotericin B and ^3^ susceptible to all antifungals.

The results for each isolate using microdilution and Etest® methods are shown in table 2 and in figure 1. Isolates from HIVpositive patients showed higher fluconazole MICs than those isolated from HIV-negative patients. Most isolates had high amphotericin B MICs, while itraconazole and ketoconazole had the lowest of the antifungal agents tested.

**Table 2:** Results from isolates according to the clinical entity for broth test and Etest^®^.

Clinical entity		Antifungal minimum inhibitory concentration (pg/ml)								
		Fluconazole		Voriconazole		Itraconazole		Amphotericin B Ketoconazole		
		M27-A3	Etest^®^	M27-A3	Etest^®^	M27-A3	Etest^®^	M27-A3	Etest^®^	M27-A3
No lesion	58.42 NL	2	2	0.25	0.064	0.125	0.25	8	3	0.25
	103.76 NL	>64	>256	>16	32	0.25	0.75	>16	32	0.5
	110.80 NL	>64	>256	4	1.5	0.25	0.25	>16	16	0.25
	9.2 NL	8	4	0.12	0.032	0.12	0.19	>16	>32	0.12
	46 NL	16	12	0.5	12	0.12	0.38	4	2	0.12
Seborrheic dermatitis	57.41 SD	4	4	2	0.094	0.062	0.25	>16	>32	0.25
	115.84 SD	>64	>256	>16	12	0.125	0.5	>16	>32	1
	2.1 SD	8	12	2	0.125	0.031	0.19	>16	6	0.25
	9.1 SD	8	12	8	0.25	0.031	0.125	>16	>32	0.5
	11.1 SD	4	12	8	0.19	0.031	0.25	>16	>32	0.25
No dermatitis and HIV	23.13 HIV	>64	>256	4	4	0.5	0.5	8	16	1
	90.64 HIV	>64	>256	8	6	0.125	0.25	16	12	0.5
	99.72 HIV	>64	>256	8	16	0.25	0.5	>16	24	0.25
	117 HIV	>64	>256	8	>32	0.5	0.38	>16	32	0.5
	40.26A HIV	4	8	2	0.125	0.062	0.38	>16	>32	0.5
Seborrheic dermatitis	24.14 SDHIV	>64	>256	4	2	0.25	0.25	16	24	0.25
and HIV	26.16 SDHIV	>64	>256	4	3	0.25	0.19	16	24	0.25
	29.18 SDHIV	8	3	0.25	0.032	0.12	0.38	4	1.5	0.063
	67.51 SDHIV	4	2	0.12	0.023	0.12	0.25	4	2	0.12
	37.248 SDHIV	2	2	0.25	0.125	0.063	0.125	4	1	0.063

M27-A3: microdilution method

### Essential agreement

**Figure 2:**
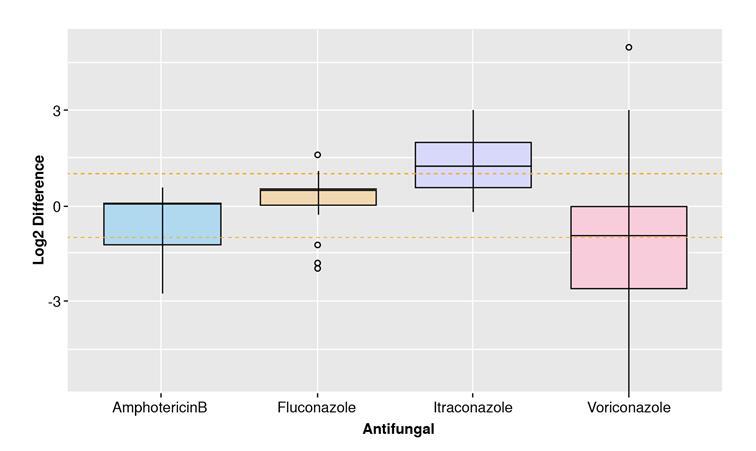
Essential agreement between MICs of Etest ® and Broth Microdilution test. Differences of the binary logarithm (Log 2) of MICs. The orange dashed lines interval shows the mean and standard deviation for each group. Both methodologies agreed in the MIC determination.

As shown in the methodology section, differences between each method’s MICs by isolate were calculated. Figure 2 shows the distribution of these values. The dotted orange lines enclose the range of the essential agreement based on its definition. Table 3 shows a results summary for each antifungal agent regarding their essential agreement and verifying the Etest^®^ method.

**Table 3:** Results of each antifungal regarding their essential agreement

Antifungal	Number of isolates within essential agreement range	Percentage of essential agreement (%)	Assumed ECV (μg/ml)	Number of isolates coinciding in classification	Percentage of categorical agreement (%)
Amphotericin B	14	70	8	18	90
Fluconazole	15	75	512	20	100*
Itraconazole	10	50	1	20	100*
Voriconazole	8	40	8	14	70

ECV: epidemiological cut-off values

* ECV for these antifungals are higher compared to the concentrations obtained or tested that all categorical interpretation are categorized in one group (none is resistant according to the ECVs).

### Categorical agreement

Based on tentative epidemiological cut-off values, isolates were classified as resistant or sensitive for each antifungal and method (table 3). Amphotericin B and voriconazole could be further analyzed, as isolates are classified as resistant and others as sensitive in each case.

Fisher’s exact test was performed to classify the isolates regarding their resistance to amphotericin B and voriconazole. A contingency table was built for each antifungal, and the p value was calculated. The null hypothesis of Fisher’s test assumes that the classification between the two methods is not different. As a result, the obtained p values for both antifungals were higher than the significance level (p>0.05), concluding that the classification of the isolates as resistant and sensitive did not differ statistically between the methods.

### Association with health conditions

**Figure 3:**
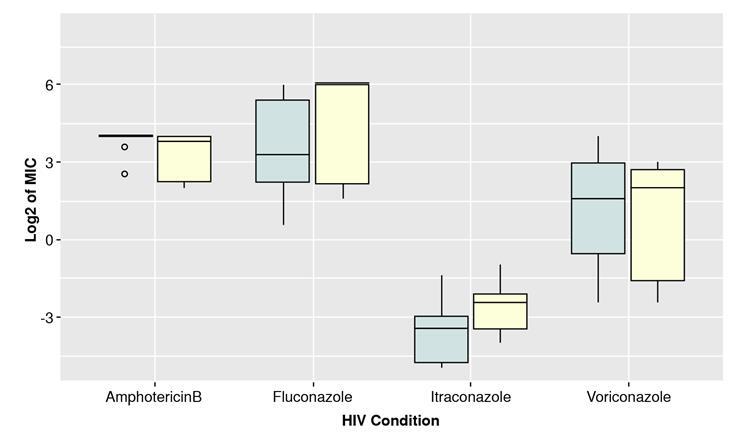
Comparison of binary logarithm (Log 2) of MICs for isolates grouped by HIV condition

**Figure 4:**
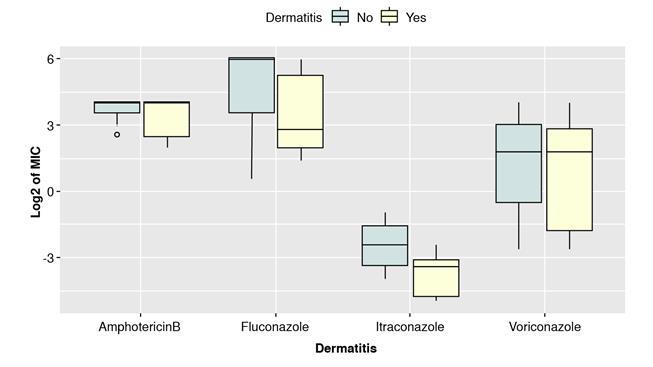
Comparison of binary logarithm (Log_2_) of MICs for isolates grouped by the presence or absence of seborrheic dermatitis

Based on the boxplots shown in figures 3 and 4, there could be a difference between the MIC of isolates from HIV-positive and negative patients and with or without dermatitis. The median of the MIC seemed to differ for each antifungal depending on the health condition, except for amphotericin B (HIV and dermatitis) and voriconazole (dermatitis). However, the interquartile ranges looked similar for the two categories in each case except for itraconazole. Shapiro-Wilk test evidenced not normally distributed data. (p>0.05). Therefore, a non-parametric test was performed (WilcoxonMannWhitney) to compare category’s means. The results indicated significant differences in MICs for itraconazole comparing isolates from patients with or without dermatitis (table 4).

**Table 4:** Results of Wilcoxon-Mann-Whitney analysis

Antifungal	HIV	Dermatitis
Amphotericin B	0.131	0.965
Fluconazole	0.498	0.176
Itraconazole	0.092	0.026
Voriconazole	0.758	0.817

## Discussion

Limited data are currently available about the *Malassezia’s* sensitivity profile. Here, we present some data to contribute to this field and, shortly, advance in collaborative studies to correlate these findings with the patient’s outcome and detect antifungal resistance.

The results show significant variability according to the clinical condition and the type of antifungal evaluated. The isolates from patients with HIV (with or without seborrheic dermatitis) have a high resistance to fluconazole (determined by both methodologies). Isolates from patients with HIV and without seborrheic dermatitis had a high MIC for voriconazole compared to those from patients with other clinical conditions. In future studies, it will be important to clarify if this resistance may be associated with the fluconazole chronic prophylactic use in people living with HIV ^37^.

On the other hand, we observed that, in general, *M. furfur* isolates showed a high MIC to amphotericin B. This finding is comparable to multiple previous studies with the same *in vitro* test results ^30^^,^^31^^,^^38^ and congruent with the prescribed treatment against *Malassezia* fungemia based on the use of amphotericin B. Some studies suggested that this effect may be associated with drug synergism or high lipid parenteral nutrition, which could increase the *in vivo* antifungal permeability of the yeast ^30^^,^^36^. Most of the isolates were sensitive to itraconazole, similar to the reported results of previous reports ^15^^,^^39^.

For the essential agreement, amphotericin B and fluconazole exhibited the closest MICs between methods (70% and 75% of essential agreement, respectively), but they could not reach the 90% to be considered comparable. Nevertheless, other studies showed essential agreement higher than 97% using supplemented Sabouraud dextrose agar ^30^.

Despite not achieving this percentage in our experiment, we suggest the use of this culture medium in the Etest® as a comparative method for sensitivity assessment ^30^. Even so, more studies are required to confirm this. The outliers in the fluconazole group could be explained by the number of isolates in the experiment. We recommend increasing the isolates sample size in future studies.

In the case of itraconazole and voriconazole, the achieved essential agreement is far below the ideal one. For itraconazole, MICs obtained using the Etest® were, on average, higher than those with the broth microdilution method. It evidenced a slight tendency of the Etest® to overestimate itraconazole’s MICs. Rhimi *et al.* reported the same finding. The authors reported a higher MIC in Etest® for the azole antifungal group, compared to the broth microdilution test using supplemented Sabouraud ^30^. Another study showed that in case of discrepancy between the methods, Etest® tended to yield higher MIC values ^29^. Likewise, this supplementation can alter the antifungal diffusion from the strip to the medium ^15^^,^^29^^,^^30^. For voriconazole, the tendency was the opposite: the Etest® method underestimated the resistance of *M. furfur* isolates.

Categorical agreement for fluconazole was 100%, but it does not validate the Etest® method because the taken epidemiological cut-off values for this antifungal had an extreme value (512 μg/ml) ^27^, so none of the isolates showed to be resistant in both cases. Determination of the categorical agreement for this antifungal agent requires an increase in the sample size that includes isolates with a wide range of MICs to differentiate resistance from sensitivity patterns.

The cut-off extreme point used for fluconazole was twice the maximum concentration evaluated in our study (512 μg/ml versus 256 μg/ml, respectively). Therefore, all our isolates apparently showed a non-resistant pattern to this antifungal. Again, due to the absence of reliable cut-off values, it is hard to define fluconazole resistance, so it will require further studies. As we mentioned, these cut-off values were taken from a previous study ^27^, meaning there are no official values to compare these nonconcordant results. Amphotericin B and voriconazole showed a possible categorical agreement comparing the microdilution with the Etest®, as previous studies reported ^29^.

Regarding the clinical condition, MICs of fluconazole, itraconazole, and voriconazole were not statistically significant for the HIV isolated strains. As for dermatitis, only fluconazole and itraconazole seemed to differ, with a higher MIC for the group without lesions. However, only itraconazole results were statistically significant. No studies have evaluated MIC in *Malassezia* isolates from HIV-positive and negative patients, but for *Candida* isolates from HIV-positive population reported an increase in MIC, possibly associated with increased exposure to antifungals as a therapeutic or prophylactic measure ^40^^-^^42^. According to the above, we could find significant differences with higher MICs for the isolates from HIV-positive patients in contrast with those from patients with dermatitis. However, the sample size, and the strains isolation place, among other factors *(e.g.,* prophylactic treatment with an antifungal agent), could have influenced the results.

In conclusion, none of the evaluated antifungal agents met the method verification. Amphotericin B was the only one that achieved compatibility. The results of this study with the supplemented Sabouraud medium presented reproducibility with those in previous reports ^30^^,^^31^^,^^38^. We found indications of differences in resistance profiles of isolates from HIVpositive patients, but further studies are needed to increase isolates sample size and confirm the findings. Finally, considering the differences between *in vitro* results and the patient’s clinical response, it is important to perform *in vivo* studies with amphotericin B in invertebrate or vertebrate models ^15^.
